# Catastrophic Antiphospholipid Syndrome and Renal Failure: An Unexpected Recovery After Three Years on Dialysis

**DOI:** 10.7759/cureus.38851

**Published:** 2023-05-10

**Authors:** Daniela Alferes, Susana Pereira, Vitória Paes de Faria, Ana Ventura, Maria Clara Almeida

**Affiliations:** 1 Nephrology, Centro Hospitalar de Vila Nova de Gaia e Espinho, Vila Nova de Gaia, PRT; 2 Nephrology, Nefrodouro Hemodialysis Clinic, Santa Maria da Feira, PRT

**Keywords:** anticoagulation, renal failure, primary antiphospholipid syndrome, catastrophic antiphospholipid syndrome, autoimmune disease

## Abstract

Catastrophic antiphospholipid syndrome (CAPS) is a rare and life-threatening disorder characterised by arterial or venous thrombotic events, involving three or more organs in a short period of time, in the presence of persistent antiphospholipid antibodies. Long-term anticoagulation with warfarin is the standard of care to prevent recurrent vascular events. Besides supportive care, optimal management of CAPS is unclear and consensus among experts is lacking. We describe a patient with primary antiphospholipid syndrome who experienced probable CAPS after receiving rivaroxaban, resulting in extensive cutaneous ulceration, acute coronary syndrome and dialysis-dependent renal failure. Anticoagulation, glucocorticoids and plasmapheresis were started. In the haemodialysis period, he maintained treatment with long-term vitamin K antagonist. The international normalized ratio target was optimized to 3.5-4. This strategy was associated with the healing of skin lesions, regression of cardiac lesions and recovery of renal function after three years on dialysis.

## Introduction

Antiphospholipid syndrome (APS) is a systemic autoimmune disorder characterised by thrombotic episodes in the arterial or venous circulation and/or pregnancy morbidity, in the presence of antiphospholipid (aPL) antibodies, namely lupus anticoagulant (LA), anticardiolipin (aCL) and anti-β2glycoprotein-I (anti-β2GPI) antibodies. APS can be either primary or secondary when it occurs in the context of other underlying autoimmune disorders, mainly systemic lupus erythematosus (SLE) [1]. Approximately 1% of APS patients develop a severe clinical scenario characterised by the development of multiple thromboses, involving three or more organs in a short period of time, usually resulting in organ failure, referred to as catastrophic APS (CAPS) [2]. When CAPS is suspected, prompt initiation of treatment is crucial. An aggressive multidisciplinary treatment strategy with anticoagulation and immunosuppression has resulted in improved patient outcomes, with a survival rate of around 50% [3-5]. Nevertheless, the long-term outcomes of patients who survive remain unclear. A study that analysed the prognosis of patients who survive the initial catastrophic event demonstrated that 66% of patients remained free from further thromboembolic events with anticoagulation during an average follow-up of 67.2 months, but 15% were functionally impaired because of CAPS, namely with end-stage cardiac failure and end-stage renal disease requiring haemodialysis [6]. We present an adult patient with end-stage kidney disease in the setting of probable CAPS, treated with long-term warfarin therapy and who remained free from further thromboembolic events and recovered kidney function after three years on dialysis.

## Case presentation

We present a 46-year-old male diagnosed with primary APS in 2004 after presenting with recurrent unprovoked deep vein thrombosis. Workup at the time revealed the presence of persistent aCL and anti-β2GPI antibodies. Past medical records included long-term hypertension treated with amlodipine 10 mg/day, dyslipidemia, cigarette smoking and chronic obstructive pulmonary disease. In December 2017, the patient developed cutaneous ulcers on both lower extremities. The ulcers were attributed to warfarin; so, the former was replaced with rivaroxaban.

One week later, the patient presented to the emergency department with a one-hour onset of chest pain. Additionally, the patient complained of severe pain in the right lower limb. On examination, his vital signs included: temperature of 36ºC, blood pressure of 150/69 mmHg, pulse rate of 87 bpm and oxygen saturation of 98% in room air. His physical examination revealed extensive necrotic skin ulcerations, mainly in the right lower extremity. The review of other systems was normal.

Laboratory workup on admission is detailed in Table 1. An electrocardiogram was obtained, which did not show any ST segment changes. Urgent bedside echocardiogram revealed mild depression of left ventricular systolic function with inferior wall hypokinesis and preserved right ventricular systolic function.

**Table 1:** Significant labs on the day of admission

Laboratory Tests	Reference Range	Patient’s Results
Haemoglobin (g/dL)	13.0-18.0	12.1
White blood count (1,000/μL)	4.4-5.9	6.84
Platelets (1,000/μL)	150-440	144
Creatinine (mg/dL)	0.67-1.17	4.4
Erythrocyte sedimentation rate (mm/hr)	0-15	102
C-reactive protein (mg/dL)	0-0.5	8.46
Troponin (ng/L)	5-14	6797.9
Brain natriuretic peptide (pg/mL)	<100	913.2

He was initially admitted to an intermediate medical unit. The cardiology emergency team assumed a provisional diagnosis of myocardial infarction, with no indication for urgent cardiac catheterization. A therapeutic dose of low molecular weight heparin was promptly administered. He remained haemodynamically stable with no recurrence of chest pain. On day 2 of admission, his platelet count (76,000/µL) and haemoglobin level (10.8 g/dL) were noticeably lower compared to the workup done on admission and his creatinine level worsened (creatinine, 4.4 mg/dL). Urinalysis showed mild proteinuria and microscopic haematuria. Renal ultrasound showed normal-sized echogenic kidneys with no hydronephrosis.

He was referred to our nephrology department for further evaluation of acute kidney injury (creatinine 5.5 mg/dL). Further workup revealed lactate dehydrogenase levels to be significantly elevated at 773 U/L (normal 0-250), a reticulocyte count of 2% and serum haptoglobin level < 6 mg/dL (normal 40-270 mg/dL). Extensive autoimmune testing looking for autoimmune disorders potentially responsible for thrombotic events was all negative. The titres of aCL and anti-β2GPI antibodies were elevated; LA was negative (Table 2). Serological testing for human immunodeficiency virus, hepatitis B virus and syphilis was negative. A renal biopsy was postponed due to thrombocytopenia and anticoagulant therapy.

**Table 2:** Results of autoimmune testing SS-A: Sjögren syndrome antigen A, SS-B: Sjögren syndrome antigen B.

Laboratory Tests	Reference Range	Patient’s Results
Rheumatoid factor antibody (UI/mL)	0-15.9	< 9.38
Antinuclear antibody (IF)	<1/160	1/160
Antimyeloperoxidase antibody (RU/mL)	0-19.9	1.2
Antiproteinase 3 antibody (RU/mL)	0-19.9	<2.0
Extractable nuclear antigen (ENA) Smith antibody (U/mL)	0-25.0	0.86
ENA nuclear riboprotein ribonucleoprotein (RNP) antibody (U/mL)	0-20.0	3.16
Anti-SS-A (Ro) (U/mL)	0-25.0	0.67
Anti-SS-B (La) (U/mL)	0-25.0	3.36
Cold agglutinins	Negative	
Protein electrophoresis	Normal	
Serum immunofixation	Negative	
C3	75-135	148
C4	9-36	29.7
Cardiolipin antibody IgG (U/mL)	Positive > 40	126.0
Cardiolipin antibody IgM (U/mL)	Negative < 10	9.5
Beta-2-glycoprotein IgG (U/mL)	Positive > 10	38.0
Beta-2-glycoprotein IgM (U/mL)	Negative < 7	7.4

The diagnosis of CAPS was considered. He was started on pulse steroid for three days, followed by prednisolone 1 mg/kg and plasmapheresis for a total of five days. Repeat testing for aCL and anti-β2GPI antibodies was performed and was reported negative after completing plasma exchange treatment. Further kidney function deterioration associated with a decrease in urine output was observed, and the patient was started on haemodialysis. He remained asymptomatic from a cardiac standpoint. Cardiac enzymes trended in the right direction. The patient was discharged from the hospital on haemodialysis three times a week, with a plan to maintain anticoagulation with warfarin with a target international normalized ratio (INR) of 3.

**Figure 1:**
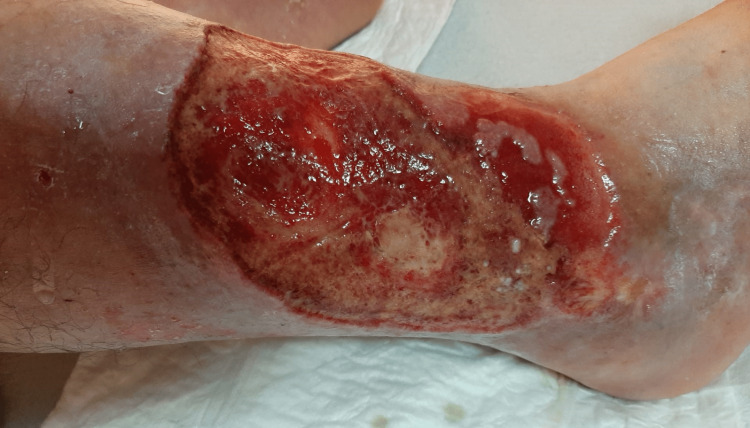
Skin lesion in the right lower extremity in November 2020 (obtained with permission from the patient).

**Figure 2:**
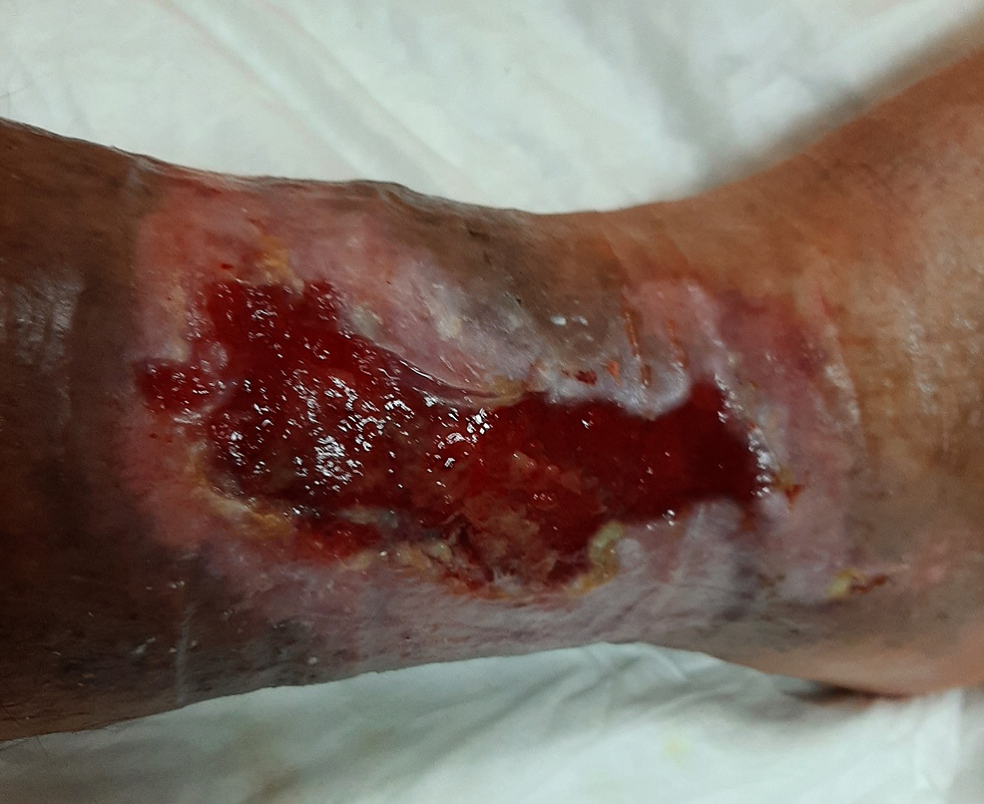
Skin lesion in the right lower extremity in March 2021 (obtained with permission from the patient).

**Figure 3:**
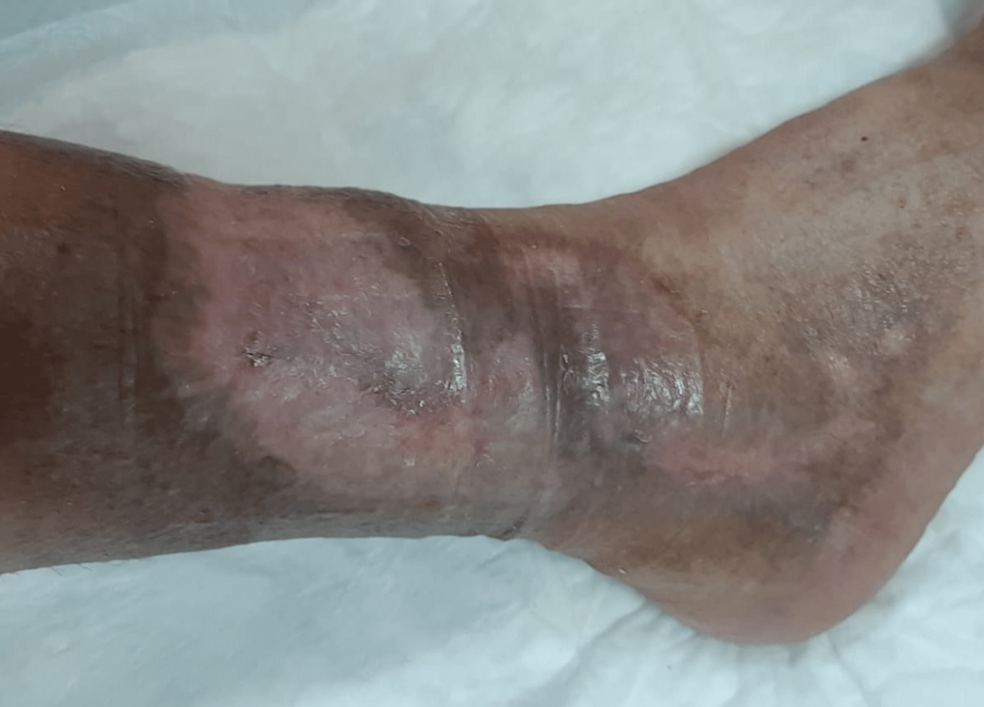
Skin lesion in the right lower extremity in July 2021 (obtained with permission from the patient).

During the dialysis period, he had arteriovenous fistula creation for haemodialysis without complications. He presented with no further episodes of chest pain but continued to have extensive chronic ulcers in the lower limbs, requiring regular treatment. After a long course of non-healing ulcers for more than a year, the medical team decided to optimize INR as high as 4 (3.5-4). The patient evolved with progressive healing of the skin lesions (Figures 1-3). A repeat transthoracic echocardiogram showed mitral valve thickening associated with mild mitral regurgitation, inferior wall hypokinesis and preserved global ventricular systolic function. Coronary angiography did not reveal obstructive disease. The patient's urine output and diuretic response improved significantly. At this time, creatinine clearance determined by 24-hour urinary creatinine excretion showed a value of 7 mL/min. The dialysis schedule was reduced to twice per week. The patient’s blood pressure remained well controlled. Stable haemoglobin (11 g/dL) allowed for erythropoietin therapy withdrawal. Lactate dehydrogenase and haptoglobin levels had normalized.

In September 2021, 45 months after the start of renal replacement, a new 24-hour clearance measurement yielded a value of 14.4 mL/min. The dialysis treatments were discontinued, and the patient has been maintained off of dialysis for the last 16 months, with a creatinine of 3.2 mg/dL. Repeat testing for aCL and anti-β2GPI antibodies was performed on April 2022 and was reported positive (46 U/mL and 55 U/mL, respectively). Repeat transthoracic echocardiogram showed preserved global ventricular systolic function, without hypokinesia of the ventricular walls as previously described.

## Discussion

This case report illustrates a complex clinical case of a 46-year-old man with a previous APS diagnosis who presented with probable CAPS. CAPS is a life-threatening condition with a mortality of over 30%, and nearly 50% when associated with lupus [3]. The clinical manifestations are quite heterogeneous and protean, reflecting the site and extent of small vessel obstruction. Registry data suggest that the major organs involved during catastrophic episodes are renal (73%), followed closely by lung (60%), brain (56%), heart (50%) and skin (47%) [4]. None of the various clinical or laboratory features is specific or pathognomonic of this condition. In order to facilitate the diagnosis, preliminary classification criteria for CAPS were proposed and later validated [7]. Additional diagnostic algorithms have been proposed to facilitate early recognition of CAPS [8]. The important steps in the proposed algorithms include evidence of simultaneous involvement of three or more organ systems within a week, confirmation by histopathology of small vessel occlusion in at least one organ or tissue, laboratory confirmation of the presence of the aPL and/or history of APS and exclusion of other causes of multiple organ thromboses or microthrombosis. In our case, multiple organ involvement (skin, heart and kidney) occurred in a short period of time, accompanied by features of thrombotic microangiopathy, in a patient with a known history of primary APS strongly suggesting probable CAPS. Histological evidence of intravascular thrombosis was not sought as the result would have not changed the patient’s management and thus expose the patient to the risks of an invasive procedure unnecessarily.

Small vessel thrombosis can have cutaneous manifestations, for example, cutaneous digital gangrene, or necrotic skin ulceration which on biopsy is secondary to diffuse non-inflammatory thrombosis [2,5,9]. Typical manifestations of cardiac disease in CAPS include valve abnormalities (thickening and vegetation), coronary artery disease, myocardial dysfunction, pulmonary hypertension and intracardiac thrombi.

Myocardial infarction occurs only 4% of the time and may be due to accelerated atherosclerosis leading to a plaque rupture or microvascular thrombosis. Autoimmune disorders, including systemic lupus erythematosus, rheumatoid arthritis, systemic sclerosis and APS, are known states of chronic inflammation that induce premature atherosclerosis [4,5]. While the involvement of aPL antibodies in the pathogenesis of thrombosis in APS is very much established in the literature, the presence of certain cardiovascular risk factors or medical conditions in these patients raises their risk for thrombosis [9]. It is noteworthy that our patient had some cardiovascular risk factors.

Kidney damage is a well-recognized complication of CAPS. Although occasionally the disease involves large vessels, in most cases, it affects small vessels, leading to a disseminated microangiopathic syndrome, resembling haemolytic uremic syndrome and thrombotic thrombocytopenic purpura, as presumed in our patient. This condition, known as APS nephropathy, is associated with a rapid decline in renal function with variable degrees of haematuria and proteinuria. It is uncertain whether antiphospholipid antibodies or other factors are implicated in the development of APS nephropathy and whether it is driven mainly by thrombotic or inflammatory processes [5,10,11]. Other glomerular lesions that have been described in patients with primary APS include membranous nephropathy, minimal change disease and pauci-immune glomerulonephritis. Although it is difficult to make conclusions on the aetiology of renal failure without renal biopsy (a limitation of this study), we consider the diagnosis of APS nephropathy more likely in this case.

The optimal management of CAPS has been a challenge since it was first described. Due to this high mortality rate, early diagnosis and aggressive treatment are essential for its successful management. Current knowledge supports the treatment with a combination of anticoagulation with heparin followed by long-term vitamin K antagonist (VKA) and high doses of glucocorticoids, as first-line treatment. Additionally, plasma exchange and/or intravenous immunoglobulins should be considered in cases with associated life-threatening situations. Intravenous cyclophosphamide is recommended in patients in which CAPS is associated with SLE. More recently, some authors have reported success in the treatment of CAPS patients refractory to conventional treatment or recurrent cases, with rituximab (monoclonal antibody against CD20 on B cells) or eculizumab (monoclonal antibody against complement component C5) [12-15]. It should be noted that this case occurred at a time when the availability of eculizumab and rituximab for these clinical situations in our centre in Portugal was limited, so these treatment options were not used at the time.

Long-term anticoagulation with VKA is the standard of care to treat and prevent a recurrent vascular event. The optimal management of patients with CAPS is less clear, and consensus among experts is lacking [5,9]. Options including standard-intensity VKA with a target INR range from 2.0 to 3.0, with or without low-dose aspirin, or higher-intensity VKA (target INR, 3.0-4.0). The INR target should be determined on an individual basis. In our patient, we decided on higher INR levels, given his high-risk profile, with recurrent episodes of thrombosis, and manifestations of CAPS. We believe that the effort to maintain INR levels at 3.5-4 in our patient was crucial for the complete healing of skin ulcers, successful recovery of renal function and prevention of future thrombotic events.

Some suggested that the intensity of the anticoagulation may be an important factor because a failure to maintain INR > 3 was associated with a more severe prognosis in patients with APS with associated renal artery stenosis [16]. A retrospective study reported the effect of anticoagulation on blood pressure control and renal function in hypertensive APS patients [17]. Fourteen of such APS patients with renal artery stenosis, who received oral anticoagulation for > 1 year, were studied retrospectively. Patients were divided into two groups based on their INR (< 3 and ≥ 3). The results suggested that patients whose INR was maintained ≥ 3 did well, their blood pressure was better controlled, renal function remained stable or improved and renal artery stenosis was reversed in some patients. In contrast, patients with a median INR < 3 had poorly controlled blood pressure and a significant deterioration in mean serum creatinine values. These findings support the idea that anticoagulation with INR maintained ≥ 3 may have an important role in preventing the progression of renal lesions associated with APS. In order to achieve the defined INR target, a mutual understanding of immunohemotherapy is crucial in order to maximize successful therapy.

One important consideration in this disorder relates to the identification of triggering factors, such as pregnancy, surgery, infection, drugs, malignancies or anticoagulation withdrawal, which may serve as a complement-amplifying “second hit” when combined with antiphospholipid antibodies and potentially a genetic variant that predisposes to complement dysregulation [2,5]. In our case, the causative role of switching anticoagulation to rivaroxaban was considered the culprit given that CAPS occurred rapidly after this treatment was started; an alternative trigger factor was not found, and the patient had been clinically stable for years with VKA as an anticoagulant treatment. Evidence about the efficacy and safety of direct oral anticoagulants (DOACs) in APS is limited. Some case reports and one randomized study published in 2019 reported that CAPS may be provoked by switching from a VKA to a DOAC [18,19]. A systematic review from 2016 documented relatively higher rates of recurrent thrombosis in individuals treated with DOACs, especially those with triple aPL positivity and/or previous arterial thrombosis [20].

## Conclusions

Since CAPS is a rare disease, case reports are important to provide a better understanding of its various clinical aspects to facilitate future diagnosis and optimize treatment. This case suggests that anticoagulation with VKA, with a defined target of INR, plays an essential role not only in preventing future thrombotic events but also in the recovery of organ manifestations, namely renal, cardiac and cutaneous, resulting from thrombotic CAPS.
